# Supplementary material for: Efficacy and safety of antimicrobial stewardship prospective audit and feedback in patients hospitalized with COVID-19: A protocol for a pragmatic clinical trial
Source: PLoS One. 2022 Mar 23;17(3):e0265493. doi: 10.1371/journal.pone.0265493 (PMC8942275; doi:10.1371/journal.pone.0265493)
Supplement: S1 File — (DOCX) [file pone.0265493.s002.docx]

**EFFICACY AND SAFETY OF ANTIMICROBIAL STEWARDSHIP INTERVENTION IN HOSPITALIZED COVID-19 PATIENTS: A PRAGMATIC CLINICAL TRIAL**

Protocol Version 6.8

Revision Date: May 21, 2021

Short name: COVASP

PROTOCOL SYNOPSIS

| Leadership and project governance | Co-principal investigators: Drs. Justin Chen, Holly Hoang, and Carlos Cervera  Division of ID Section Chief: Dr. Karen Doucette  UAH ASP team: Cecilia Lau, RPh, Jackson Stewart, RPh, Dr. Stephanie Smith, Dr. Dima Kabbani  GNH ASP team: Karen Zurek, RPh  MCH ASP team: Morgan Schultz, RPh, Shahileen Remtulla, RPh  Biostatistician: Maryna Yaskina, PhD |
| --- | --- |
| Overview | Prospective audit and feedback in patients hospitalized with acute COVID-19. |
| Design and Setting | Prospective, multi-center, non-inferiority pragmatic clinical trial evaluating prospective audit and feedback of antimicrobials in patients hospitalized with COVID-19.  Sites enrolled:  1) University of Alberta Hospital and Mazankowski Alberta Heart Institute, Edmonton, Alberta  2) Grey Nuns Community Hospital, Edmonton, Alberta  3) Misericordia Community Hospital, Edmonton, Alberta |
| Study Population | Adult patients (≥18 years old) diagnosed with and admitted to hospital with acute COVID-19. |
| Intervention | Antimicrobial stewardship prospective audit and feedback. Intervention will be applied patients admitted to pre-randomized hospital beds. |
| Primary Objective | To determine if antimicrobial stewardship intervention is non-inferior to no antimicrobial stewardship intervention on clinical outcomes at 15 days of hospitalization in patients admitted with acute COVID-19. |
| Secondary Objectives | To determine if an antimicrobial stewardship intervention reduces the length of hospitalization, mortality, re-admission rates, antimicrobial utilization, rate of multi-drug resistant infections, and the rates of adverse events. |
| Primary Endpoint | A 7 point ordinal scale of clinical outcomes, measured at day 15 from hospital admission. |
| Secondary Endpoints | Clinical:  Length of hospital stay  In-hospital mortality  30 day mortality  *C. difficile* associated mortality  30 day readmission rate  Antimicrobial Stewardship:  Days of therapy  Length of therapy  Number of audits  Types of recommendations  Acceptance rate  Microbiologic:  *C. difficile* associated diarrhea  Infection with multi-drug resistant bacteria  Adverse events/complications:  Neutropenia  Acute kidney injury |
| Statistical analysis | The primary outcome will be a two-sample comparison of scores between the treatment and control arm. We will assess whether the scores in the treatment arm are not worse than in the control arm using the Mann-Whitney U test. A one-sided level of 0.025 will be used to declare significance for the non-inferiority. Results will be reported along with 95% CI. |
| Timeline | Enrollment is estimated to commence March 1, 2021. The study will be approximately 6-12 months in duration. |
| Sponsorship | Unfunded |

TABLE OF CONTENTS

1. BACKGROUND AND RATIONALE
   1. COVID-19 pandemic
   2. Coinfections and antimicrobial therapy
   3. Antimicrobial stewardship programs
   4. Antimicrobial stewardship in COVID-19
2. OBJECTIVES
3. EVALUATION QUESTIONS
   1. Clinical evaluation primary questions
   2. Clinical evaluation secondary questions
   3. Antimicrobial stewardship primary questions
4. METHODS
   1. Study design
   2. Participating centers
   3. Eligibility criteria
      1. Population
      2. Inclusion criteria
      3. Exclusion criteria
   4. Project protocol
      1. Sample size estimation
      2. Interventions
         1. Antimicrobial stewardship program (ASP)
5. PROJECT ENDPOINTS
   1. Primary endpoint measures
   2. Secondary endpoint measures
      1. Clinical endpoints
      2. Antimicrobial stewardship endpoints
      3. Microbiologic endpoints
      4. Adverse events/complications
6. DATA COLLECTION, SOURCES, AND MANAGEMENT
7. STATISTICAL ANALYSIS
8. REGULATORY AND ETHICAL STANDARDS
   1. Ethical considerations
   2. Maintenance of records
   3. Confidentiality
9. APPENDICES
10. REFERENCES

1. **BACKGROUND AND RATIONALE**
   1. **COVID-19 pandemic**

COVID-19 is respiratory disease caused by the severe acute respiratory coronavirus 2 (SARS-CoV-2), a novel coronavirus which has spread rapidly across the world with over 149.9 million laboratory confirmed cases and over 3.1 million reported deaths since December 2019 (<https://coronavirus.jhu.edu/map.html>, accessed April 29, 2021). As the COVID-19 pandemic continues there is an increasing number of patients hospitalized and requiring care in strained healthcare systems. Studies report an average mortality rate of 2% in the general population and as high as 14% in the ICU population. There are limited mortality-altering strategies to manage COVID-19. Vaccine is early stages of delivery and majority of experts and officials across the world do not anticipate an end to the pandemic in the imminent future. Faced with the pressure of this pandemic, high quality data with randomized controlled trials are needed to support various COVID-19 management guidelines.

- 1. **Coinfections and antimicrobial therapy**

Secondary bacterial infections are known complications of viral pneumonia in influenza and other respiratory viruses^1,2^. It is estimated that approximately 4-8% of hospitalized patients with COVID-19 have co-infection with bacterial pathogens^3,4^. The microbiology appears to be heterogeneous ranging from gram positive to gram negative bacteria, and even polymicrobial pneumonias.

However, there is significant and widespread antibiotic use in hospitalized patients with COVID-19 reported in the literature. Most cohort studies report a range from 40-70%^5-9^, however some studies report up to 100% of hospitalized patients with COVID-19 receive antibacterials^8,10,11^. In these reports, antibiotics prescribed were often broad-spectrum^9,12-14^ including respiratory fluoroquinolones, cephalosporins, and carbapenems.

There is a large disconnect between the prevalence of proven bacterial co-infections and the rate of antibiotic utilization in patients with COVID-19.

- 1. **Antimicrobial stewardship programs**

Antimicrobial Stewardship Programs (ASP) is an Accreditation Canada Required Organizational Practice. Its role is to review antimicrobial use and management of infectious diseases systematically to ensure that antimicrobial prescribing is appropriate, with the goal of slowing the development of antimicrobial resistance, optimizing patient outcomes, and improving efficiencies. ASPs employ a multidisciplinary team involving infectious disease physicians, pharmacists, infection prevention and control programs, the microbiology lab, hospital administration, and information technology specialists, amongst others.

There are numerous ASP intervention strategies that have been described to be effective. Prospective audit and feedback (PAF), restriction and pre-authorization, formulary review, education, clinical practice guidelines, clinical pathways for antimicrobial prescribing, order sets, strategies for streamlining or de-escalation of therapies, dose optimization, and parenteral to oral conversion of antimicrobials are such examples.

One of the most effective and well described ASP strategies is PAF where antimicrobial stewardship teams review patients' charts and provide real-time prescribing feedback to attending teams to optimize antimicrobial therapy. This is a post-prescription strategy that sets to course-correct suboptimal prescribing and is meant to be collaborative in nature. It serves as a clinical service which provides education and recommendations based on an individual patient’s clinical context without providing direct clinical care. In other words, there is no interaction with the patient before, during, or after the intervention.

- 1. **Antimicrobial stewardship in COVID-19**

The majority of COVID-19 management guidelines recommend judicious use of antimicrobials in patients presenting with pneumonia. *Clostridioides difficile* outbreaks have been described to be related to often excessive antimicrobial therapy for community acquired pneumonia^15^. Additionally, antimicrobials can be associated with adverse drug events such as allergy, acute kidney injury, or ventricular arrhythmias related to long QT syndrome^16^.

Given the discrepancy between estimated coinfection rates and antimicrobial prescribing rates, calls for antimicrobial stewardship involvement in managing the COVID-19 pandemic have been made. Many have raised concerns in patients with COVID-19 given the widespread use of antibiotics reported in descriptive series and have highlighted the crucial role of formal antimicrobial stewardship programs in combating antimicrobial resistance and antimicrobial related complications^3,17,18^.

While the benefit and safety of antimicrobial stewardship interventions is established in other settings, many centers and jurisdictions do not have ASP or systematic ASP interventions. Our study aims to determine the benefits and safety of ASP intervention in patients hospitalized with acute COVID-19.

1. **OBJECTIVE**

The objective of this study is to determine the effect of an antimicrobial stewardship intervention (prospective audit and feedback) on clinical outcomes in patients hospitalized with acute COVID-19.

1. **EVALUATION QUESTIONS**
   1. **Primary clinical evaluation questions:**
2. Does antimicrobial stewardship intervention negatively impact clinical outcomes at 15 days using a 7-point ordinal scale in hospitalized patients with acute COVID-19?
   1. **Clinical evaluation secondary questions:**
3. Does antimicrobial stewardship intervention improve length of stay, mortality, or readmission rates in patients with acute COVID-19?
   1. **Antimicrobial stewardship questions:**
4. Does antimicrobial stewardship intervention result in decreased antibiotic utilization in patients with acute COVID-19?
5. How often ASP interventions are required (i.e. # interventions/number of COVID-19 patients audited)?
6. What is the rate of acceptance to prospective audit and feedback recommendations?
7. What is the rate of multi-drug resistant and *C. difficile* infections?
8. **METHODS**
   1. **Study design**

This is a prospective, multi-center, non-inferiority pragmatic trial of prospective audit and feedback versus no antimicrobial stewardship intervention in patients with proven SARS-CoV-2 infection microbiologically confirmed in the preceding 2 weeks and resulting hospital admission due to acute COVID-19.

Each participating hospital will generate a line list of all hospital beds in adult COVID units and critical care units in advance of enrollment. The line list will include the designated native beds in each room on the unit and additional theoretical “surge beds” per room in the case of hospital overcapacity taking into account the potential for surge during the pandemic. The line list of beds will be stratified by COVID unit and critical care unit beds, and will be computer randomized in a 1:1 fashion into 2 arms (ASP intervention versus no ASP intervention) prior to study commencement at the participating site.

If additional COVID or critical care units at a participating hospital are opened in the event of pandemic surge, the beds within the newly opened units will be included in the line list and the entire bed line list of the participating hospital will be subsequently re-randomized using the same randomization rules, to ensure appropriate stratification to COVID units and critical care units.

- 1. **Participating centers**

Participating centers as of May 21, 2021 include:

1. University of Alberta Hospital (including the Mazankowski Alberta Heart Institute), Edmonton, Alberta
2. Grey Nuns Community Hospital, Edmonton, Alberta
3. Misericordia Community Hospital, Edmonton, Alberta
   1. **Eligibility criteria**
      1. **Inclusion criteria**

All hospital beds in designated COVID units and critical care units accepting adult patients with confirmed acute COVID-19 pneumonia at a participating hospital will be pre-randomized.

Patients are eligible for enrollment in the study if they meet all of the following inclusion criteria:

1. Age ≥ 18 years at the time of hospital admission.
2. Confirmed SARS-CoV-2 infection by nucleic acid testing in the preceding 14 days of hospital admission.
3. Admitted from the community (including continuing care facilities).
4. They are admitted to a hospital bed designated in the study.
5. They have a SpO2 ≤ 94% on room air, or requiring supplemental oxygen, or chest imaging findings compatible with COVID-19 pneumonia.
   - 1. **Exclusion criteria**

All hospital beds outside of designated COVID units and critical care units will be excluded from randomization.

A patient will be excluded from the study if:

1. The patient is enrolled in another clinical trial that involves antibacterial therapy.
2. The patient’s goals of care is anticipated to be designated “total compassionate care” or palliative care within 48 hours of admission.
3. The patient’s progression to death is anticipated to be imminent and inevitable within 48 hours of admission.
4. The patient was attended by any member of the research team within 30 days of enrollment.
5. The patient is transferred from another acute care center.
   1. **Antimicrobial stewardship intervention**

The antimicrobial stewardship intervention employed will be prospective audit and feedback (PAF). That is an unsolicited review of active antimicrobial prescriptions with real time feedback to attending teams (i.e., the most responsible attending physician). ASP teams do not interact with the patient or their family members at any time before, during, or after the study period. Audits are performed prospectively, on weekdays less statutory holidays, by members of the ASP team consisting of infectious disease or antimicrobial stewardship physicians or pharmacists. Verbal and written feedback (i.e., appendix 9.4) will be provided in real time to attending team members (most often the attending physician) if the antimicrobial stewardship team is making a specific recommendation.

The initial PAF will occur on the day an eligible patient is identified and enrolled in the study. Follow-up audits will then occur weekly (+/-3 days to account for weekends or statuary holidays) and ad-hoc if a new antibacterial is prescribed, until the primary end-point at post-admission day 15. Appropriateness in antimicrobial prescribing will be assessed based on local clinical practice guidelines (i.e., COVID-19 Scientific Advisory Group recommendations^19^). If no such guidelines exist, then appropriateness is defined by expert opinion of the antimicrobial stewardship team member performing the audit. The focus of ASP recommendations will be to optimize the duration of therapy, to discontinue therapy where no bacterial secondary infection is found, and to optimize the spectrum of antimicrobials prescribed.

Patients with COVID-19 will be identified by the Alberta Health Services Tableau dashboard, direct notification from site-based Infection Prevention & Control Programs, direct screening of COVID or critical care units, and/or notification through Northern Alberta Clinical Trials + Research Center (NACTRC).

Only antibacterials will be audited. Antimycobacterial, antiviral, antifungal, antiparasitic agents will not be audited. Prescriptions will be excluded from PAF if they are single doses or discontinued prior to PAF. Prescriptions will be also be excluded from PAF and the final analysis if being used for surgical or medical prophylaxis.

Patients will be followed and analyzed in the arm they were assigned to regardless of transfers or movements through the hospitalization period. Patients will be followed out to 15 days where the primary endpoint will be assessed, and then up to 30 days for assessment of secondary endpoints.

- 1. **Sample size estimation**

A total of 530 patients (265 per arm) are needed to show a statistically significant non-inferiority with 80% power and 2.5% one-sided alpha assuming standard deviation of 2 and the non-inferiority margin of 0.5. Accounting for 5% drop out rate, 279 participants will be recruited in each arm (558 participants total).

When 250 participants from the control arm reach 15 days follow up or when 260 patients are recruited in the control arm (whichever is earlier) a non-comparative sample size reassessment will be performed. The standard deviation will be calculated for the control group and will be used to recalculate the sample size.

1. **PROJECT ENDPOINTS**
   1. **Primary endpoint measures**

A 7 point ordinal scale of clinical outcomes, measured at day 15 from hospital admission.

| **Clinical Outcome** | **Points** |
| --- | --- |
| Not hospitalized, able to resume normal daily activities | 1 |
| Not hospitalized, unable to resume normal daily activities | 2 |
| Hospitalized, not on supplemental oxygen | 3 |
| Hospitalized, on supplemental oxygen | 4 |
| Hospitalized, on high flow oxygen therapy or non-invasive mechanical ventilation | 5 |
| Hospitalized, on ECMO or invasive mechanical ventilation | 6 |
| Death | 7 |

- 1. **Secondary endpoint measures**
     1. **Clinical endpoints**

Hospital length of stay, in-hospital and 30 day mortality, *C. difficile* associated mortality, and 30 day re-admission rates will be examined.

- - 1. **Antimicrobial stewardship endpoints**

Days of therapy for the duration of hospitalization (capped at 30 days) of specific antimicrobial utilization normalized for patient-days and length of total antimicrobial therapy normalized for patient-days will be examined. Furthermore, the number of audits, types of recommendations, and rate of acceptance will be determined.

- - 1. **Microbiologic endpoints**

The 30 day multi-drug resistant infection rates and 30 day *C. difficile* infection rate will be examined.

- - 1. **Adverse events/complications**

The 30 day rates of neutropenia and acute kidney injury as diagnosed and staged as according to KDIGO will be examined.

1. **DATA COLLECTION, SOURCES, AND MANAGEMENT**

Process and outcome measures are prospectively captured by site antimicrobial stewardship teams for quality improvement purposes. These data elements will include baseline patient demographics, comorbidities, date of hospital admission, infection source (i.e., community-acquired vs hospital-acquired), microbiology results (sputum culture, respiratory pathogen panels, and blood cultures), physiology (i.e., oxygen requirements), laboratory, chest imaging findings, antimicrobial therapy details, concomitant COVID-19 therapies (i.e., dexamethasone), and ASP intervention details and outcomes. Additional Sequential Organ Failure Assessment (SOFA) score (along with its 6 individual components) and intensity of support (i.e., mechanical ventilation) data are collected in patients admitted to intensive care units.

In-hospital and 30 day mortality, length of stay, and 30-day readmission rates will be requested from pre-existing databases (Communicable Disease & Outbreak Management database, Discharge Abstract Database). Thirty-day multi-drug resistant pathogen infection and *C. difficile* infection rates will be requested from the provincial Infection Prevention and Control database (ProvSurv). Antimicrobial stewardship outcomes will be captured including antimicrobial utilization data in the form of days of therapy of each individual antimicrobial, and total length of therapy during hospitalization. Each prescription will be audited with respect to its indication and appropriateness. The rationale for antimicrobial stewardship recommendations, if any, will be recorded.

The complete schedule of data variables and data collection form is available as an appendix, items 9.2 and 9.3.

1. **STATISTICAL ANALYSIS**

All analyses will adhere to the principle of intention-to-treat (ITT). The ITT population will include all participants who were randomized in the trial. Additional analyses will be conducted on the per-protocol (PP) populations. The PP population will include all patients who completed the study as described in the protocol. This additional analysis will only be presented if there is a substantial difference in this populations compared to the ITT population.

The primary outcome will be a two-sample comparison of scores between the treatment and control arm. We will assess whether the scores in the treatment arm are not worse than in the control arm using the Mann-Whitney U test. A one-sided level of 0.025 will be used to declare significance for the non-inferiority. Results will be reported along with 95% CI.

Binary outcomes will be analyzed by a two-sample comparison of proportions using chi-square test. Continuous variables will be tested either by the student’s t test or by the Wilcoxon rank sum test depending whether assumptions for the t-test are satisfied.

Fisher exact test will be used to determine the statistical significance of difference with respect to the incidence of serious adverse events between the treatment and control arms.

Baseline characteristics will be presented by the appropriate descriptive statistics: continuous variables will be summarized by mean, standard deviation, median, quartiles, minimum and maximum. Categorical data will be presented by absolute and relative frequencies (n and %).

All subgroup analyses will be considered exploratory. Comparison of the outcomes by sex (male/ female), age group (by median age), and comorbidities will be performed. This analysis will be planned and described in SAP.

Primary and secondary outcomes will be adjusted for covariates. All adjusted analysis will be exploratory. Co-variates of interest will be included based on clinical relevance and will be specified in SAP. Adjustment will be performed by adding covariates to the original models.

1. **REGULATORY AND ETHICAL STANDARDS**
   1. **Ethical considerations**

Antimicrobial stewardship programs and its interventions are an Accreditation Canada Required Organizational Practice. Its interventions are typically classified as quality improvement initiatives targeting antibiotic prescribing. One specific ASP intervention strategy, prospective audit and feedback of specific antimicrobials or infectious syndromes, is an unsolicited clinical education/reminder service that is considered standard of care for many centers due to their benefit. However, utility and safety of prospective audit and feedback has not been definitively proven for patients with acute COVID-19.

This study was reviewed by the University of Alberta Research Ethics Board with an application submitted to the Alberta Research Information Services (Pro00105598).

A waiver of consent pursuant to [Article 3.7 of TCPS2](https://ethics.gc.ca/eng/tcps2-eptc2_2018_chapter3-chapitre3.html#b) was requested with the following justification:

1. *The research involves no more than minimal risk to the participants.*

This study involves no more than minimal risk to participants. First, neither the research team nor the antimicrobial stewardship team will ever interact with patients, their families, or their delegates at any point before, during, or after the study. Secondly, any antimicrobial stewardship recommendations through prospective audit and feedback must be reviewed and approved by the most responsible physician (attending physician) before any changes to the clinical care for the patient are executed. In other words, prospective audit and feedback serves as a focused, education and reminder service contextualized to the individual patient, but does not provide any direct clinical care to patients.

1. *The alteration to consent requirements is unlikely to adversely affect the welfare of participants.*

It is highly unlikely that alterations to consent requirements will adversely affect the welfare of participants. Any antimicrobial stewardship recommendations through prospective audit and feedback must be reviewed and approved by the most responsible physician (attending physician) before any changes to the clinical care for the patient are executed. In other words, prospective audit and feedback serves as a focused education and reminder service contextualized to the individual patient, but does not provide any direct clinical care to patients.

1. *It is impossible or impracticable (see Glossary) to carry out the research and to address the research question properly, given the research design, if the prior consent of participants is required.*

It is impracticable to carry out the research and to address the research question properly if physician consent is obtained as if the most responsible physician is aware of his/her medical practice being monitored, prescribing behaviors will immediately change (i.e., the Hawthorne effect) and therefore jeopardizing the validity of results^20^.

1. *In the case of a proposed alteration, the precise nature and extent of any proposed alteration is defined.*

In the case of a proposed alteration, the precise nature and extent of any proposed alteration will be defined.

1. *The plan to provide a debriefing (if any) that may also offer participants the possibility of refusing consent and/or withdrawing data and/or human biological materials, shall be in accordance with Article 3.7B.*

There will be no debrief.

- 1. **Maintenance of records**

The research team will maintain a password-protected, encrypted master list linking minimal identifiers (names, healthcare number) to a unique study identification number to enable records to be found at a later date. The master list will be password-protected, encrypted, and stored on an Alberta Health Services or Covenant Health restricted-access network drive. De-identified data will be collected and stored in REDCap.

The project personnel will maintain all records, patient files, and other source data for the time periods required by regulatory authorities.

- 1. **Confidentiality**

All patient information will be stored on a high security computer system and kept strictly confidential. Patient medical information obtained as a result of this study is considered confidential and disclosure to third parties is prohibited.

The research team will maintain a password-protected, encrypted master list linking minimal identifiers (names, healthcare number) to a unique study identification number to enable records to be found at a later date. The master list will be password-protected, encrypted, and stored on an Alberta Health Services or Covenant Health restricted-access network drive. De-identified data will be collected and stored in REDCap.

The project personnel will maintain all records, patient files, and other source data for the time periods required by regulatory authorities.

1. **APPENDICES**
   1. **Line list of eligible beds for enrollment**
      1. **University of Alberta Hospital**

UAH bed line list 2021-02-11.xlsx

- - 1. **Grey Nuns Community Hospital**

GNH bed line list 2021-02-12.xlsx

- - 1. **Misericordia Community Hospital**

MCH bed line list 2021-02-12.xlsx

- 1. **Schedule of data variables**

COVASP Database Blank v5.1.xlsx

- 1. **Data collection form**

COVASP Data collection form.docx

- 1. **Antimicrobial stewardship PAF written feedback example form**

PAF Example Written Feedback 2020-12-17.docx

1. **REFERENCES**

1. MacIntyre CR, Chughtai AA, Barnes M, et al. The role of pneumonia and secondary bacterial infection in fatal and serious outcomes of pandemic influenza a(H1N1)pdm09. BMC Infect Dis 2018;18:637.

2. Shah NS, Greenberg JA, McNulty MC, et al. Bacterial and viral co-infections complicating severe influenza: Incidence and impact among 507 U.S. patients, 2013-14. J Clin Virol 2016;80:12-9.

3. Rawson TM, Moore LSP, Zhu N, et al. Bacterial and fungal co-infection in individuals with coronavirus: A rapid review to support COVID-19 antimicrobial prescribing. Clin Infect Dis 2020.

4. Langford BJ, So M, Raybardhan S, et al. Bacterial co-infection and secondary infection in patients with COVID-19: a living rapid review and meta-analysis. Clin Microbiol Infect 2020;26:1622-9.

5. Chen N, Zhou M, Dong X, et al. Epidemiological and clinical characteristics of 99 cases of 2019 novel coronavirus pneumonia in Wuhan, China: a descriptive study. Lancet (London, England) 2020;395:507-13.

6. Feng Y, Ling Y, Bai T, et al. COVID-19 with Different Severity: A Multi-center Study of Clinical Features. American journal of respiratory and critical care medicine 2020.

7. Wan S, Xiang Y, Fang W, et al. Clinical features and treatment of COVID-19 patients in northeast Chongqing. Journal of medical virology 2020.

8. Wang Z, Yang B, Li Q, Wen L, Zhang R. Clinical Features of 69 Cases with Coronavirus Disease 2019 in Wuhan, China. Clin Infect Dis 2020.

9. Zhao XY, Xu XX, Yin HS, et al. Clinical characteristics of patients with 2019 coronavirus disease in a non-Wuhan area of Hubei Province, China: a retrospective study. BMC Infect Dis 2020;20:311.

10. Cao J, Hu X, Cheng W, Yu L, Tu WJ, Liu Q. Clinical features and short-term outcomes of 18 patients with corona virus disease 2019 in intensive care unit. Intensive Care Med 2020.

11. Huang C, Wang Y, Li X, et al. Clinical features of patients infected with 2019 novel coronavirus in Wuhan, China. Lancet 2020;395:497-506.

12. Barrasa H, Rello J, Tejada S, et al. SARS-Cov-2 in Spanish Intensive Care: Early Experience with 15-day Survival In Vitoria. Anaesthesia Critical Care & Pain Medicine 2020.

13. Wang D, Hu B, Hu C, et al. Clinical Characteristics of 138 Hospitalized Patients With 2019 Novel Coronavirus-Infected Pneumonia in Wuhan, China. JAMA 2020.

14. Du Y, Tu L, Zhu P, et al. Clinical Features of 85 Fatal Cases of COVID-19 from Wuhan: A Retrospective Observational Study. American Journal of Respiratory and Critical Care Medicine 2020.

15. Polgreen PM, Chen YY, Cavanaugh JE, et al. An outbreak of severe Clostridium difficile-associated disease possibly related to inappropriate antimicrobial therapy for community-acquired pneumonia. Infect Control Hosp Epidemiol 2007;28:212-4.

16. Sapp JL, Alqarawi W, MacIntyre CJ, et al. Guidance On Minimizing Risk of Drug-Induced Ventricular Arrhythmia During Treatment of COVID-19: A Statement from the Canadian Heart Rhythm Society. The Canadian journal of cardiology 2020.

17. Clancy CJ, Nguyen MH. COVID-19, superinfections and antimicrobial development: What can we expect? Clin Infect Dis 2020.

18. Stevens MP, Patel PK, Nori P. Involving antimicrobial stewardship programs in COVID-19 response efforts: all hands on deck. Infection Control & Hospital Epidemiology 2020:1-6.

19. Current Guidance for the Management of Adult Hospitalized Patients with COVID-19. Alberta Health Services, 2020. (Accessed December 17, 2020, 2020, at <https://www.albertahealthservices.ca/assets/info/ppih/if-ppih-covid-19-recommendations.pdf>.)

20. Chen LF, Vander Weg MW, Hofmann DA, Reisinger HS. The Hawthorne Effect in Infection Prevention and Epidemiology. Infect Control Hosp Epidemiol 2015;36:1444-50.
